# Neuromodulation techniques in combination for a short-intensive treatment of depression and anxiety: a case report

**DOI:** 10.3934/Neuroscience.2025009

**Published:** 2025-05-14

**Authors:** Fiammetta Iannuzzo, Federica Donia, Lorenzo Bette, Fabrizio Turiaco, Antonio Bruno

**Affiliations:** Department of Biomedical and Dental Sciences and Morphofunctional Imaging, University of Messina, Via Consolare Valeria 1, Contesse, Messina 98125, Italy

**Keywords:** depression, anxiety, rTMS, tDCS, neuromodulation

## Abstract

Neuromodulation techniques have emerged as valuable strategies for patients with depression and anxiety who do not respond to traditional therapies. Repetitive transcranial magnetic stimulation (rTMS) and transcranial direct current stimulation (tDCS) treatments are heterogeneous; however, they all share an average duration of at least 10 days, thus requiring significant patient commitment to maintain adequate compliance. Here we describe a 68-year-old woman who suffered from depression and generalized anxiety disorder and underwent a short-intensive combined protocol, thus highlighting its effectiveness and good tolerability.

## 1. Introduction

The prevalence of depression and anxiety disorder has shown a marked increase in the last years, thus highlighting their status as a growing worldwide public health issue [1]. Depression is characterized by persistent feelings of sadness, hopelessness, and a loss of interest or pleasure in activities, and is often accompanied by physical symptoms such as changes in appetite, sleep disturbances, and fatigue [2], and behavioral symptoms, which cover emotional, motivational, cognitive, and physiological domains, and include anhedonia and memory alterations [3]. Anxiety disorders involve excessive worry, fear, and physiological symptoms such as increased heart rate and restlessness, insomnia, and dizziness [4],[5]. Their treatment typically includes pharmacological approaches, such as antidepressants (e.g., selective serotonin reuptake inhibitors) and anti-anxiety medications, as well as psychological interventions such as cognitive-behavioral therapy (CBT), which focuses on restructuring negative thought patterns, and mindfulness-based interventions, which help individuals develop awareness and an acceptance of their thoughts and feelings. In this field, neuromodulation techniques, such as transcranial magnetic stimulation (TMS) and transcranial direct current stimulation (tDCS), have emerged as valuable strategies for patients who do not respond to traditional therapies, thus offering promising results in managing these debilitating conditions.

Repetitive TMS (rTMS) uses magnetic pulses that penetrate the scalp and reach the brain through a coil placed over a patient's head, and is based on the activation of neurons by depolarization [6]. It is usually used for both psychiatric and neurological disorders through inhibitory (1 Hz and continuous theta burst stimulation) or excitatory (>5 Hz and intermittent theta burst stimulation) protocols [7], with different levels of evidence according to the most recent guidelines [8]. In the treatment of depression and anxiety, the dorsolateral prefrontal cortex (DLPFC) is an area of major interest, mostly identified using neuronavigation systems [9]. Otherwise, tDCS uses two electrodes (one anode and one cathode) which create a feeble continuous current (1–2 mA) and acts on cortical excitability [10]. It seems to be effective for the treatment of unipolar and bipolar depression, with effects for active versus sham tDCS considered small-to-medium in depressive symptoms [11],[12], and may also alleviate chronic anxiety disorders [13].

However, longer protocols demand a greater time investment and commitment from patients, and often leads to a poor compliance or logistical challenges that hinder the treatment completion. For this reason, achieving significant results within a shorter timeframe would be highly beneficial for patients.

Given this background, we decided to use a short and more intensive protocol of only 5 days that can act on both depression and anxiety symptoms. The distinctive feature of the protocol lies in the short timeframe and in the sequential application of combined tDCS and TMS techniques, aiming to maximize the neuromodulatory effect. rTMS induces synaptic plasticity in a frequency-dependent manner, thus producing effects similar to long-term potentiation (LTP) or depression (LTD); on the other hand, tDCS modulates the baseline excitability of neurons, which enhances it with anodal or cathodal stimulation, thereby making neural circuits more responsive to subsequent interventions. In this context, tDCS can prime the cortex, thus creating a more favorable state for TMS to induce stronger and more targeted neuroplastic changes [14],[15].

## 2. Case presentation

G. was a 68-year-old woman who suffered from depression and generalized anxiety disorder. Her psychopathological onset dates were recorded about 30 years ago and it was characterized by sadness and anxiety symptoms. From 2006 to 2015, a neurologist specialist prescribed prazepam and paroxetine for her; however, she had a poor compliance with pharmacological treatment over the years. She either decreased or stopped using the drugs on her own. She had a period of total remission (2015) which was interrupted by a cancer diagnosis. Over the last 10 years, there were periods of well-being alternating with periods of worsening symptoms.

The therapies prescribed during these years were as follows: delorazepam, citalopram, paroxetine, and venlafaxine. At the time the patient arrived at our observation, she was in a treatment comprised of duloxetine 30 mg/day, quetiapine 100 mg/day (slow-release formulation), and delorazepam 1 mg/day. She was referred to the neuromodulation clinic due to the persistence of mild symptoms despite the pharmacotherapy. The patient provided written informed consent after an explanation of the protocol design.

The protocol developed for her consisted of the combination of tDCS and rTMS (and for this reason we considered it intensive), and included two sessions/day separated by a 20-minute break for five consecutive days.

The first session provided anodal tDCS stimulation at the level of the left DLPFC (lDLPFC) followed by rTMS stimulation of the right DLPFC (rDLPFC). The brain areas were identified by locating landmarks through craniometric references, without the aid of magnetic resonance imaging. Specifically, the tDCS parameters were as follows: electrodes (PiSTIM) with a diameter of 12 mm and a circular surface area of approximately 3.14 cm^2^. The electrodes were placed at the DLPFC. Specifically, anodal stimulation was performed with a voltage of 2000 µA, at the level of the lDLPFC, and cathodic stimulation was performed on the corresponding area on the rDLPFC. A highly conductive saline gel was applied to each electrode, and the protocol lasted for 20 min and 4 seconds. The second session provided the same tDCS treatment as the first one but was followed by rTMS stimulation of the lDLPFC. The r-TMS protocol of the rDLPFC consisted of a frequency of 1Hz (parameters: 600 stimuli/session, 30 pulses/train, 20 total trains, 1 s inter-train interval at 80% motor threshold (MT) for the hand muscles); the rTMS protocol of the lDLPFC consisted of a frequency of 10Hz (parameters: 3000 stimuli/session,40 pulses/train,75 total trains, 26 s inter-train interval at 110% MT).

**Figure 1.**
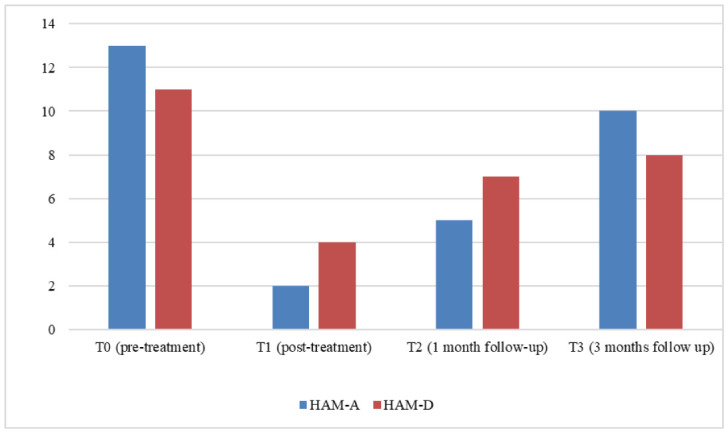
Clinical assessment at baseline (T0), at the end of treatment (T1), after 1 month (T2), and after 3 months (T3).

The Hamilton Anxiety Scale (HAM-A) and the Hamilton rating scale for depression (HAM-D) were used for the clinical assessment [16],[17]. These scales were administered twice to the patient: once before the beginning of treatment (T0) and once at the end of the treatment (T1). At T0, the score was indicative of mild anxiety (HAM-A score = 13) and mild depression (HAM-D score = 11). At T1, the scores indicated no depression or anxiety (HAM-A score = 2; HAM-D score = 4), which suggests that the patient reported remission of both the depression and anxiety symptoms (see Figure 1). Moreover, the tDCS/rTMS combination was well tolerated; the only one side effect was a slight headache after the first day of treatment, with a remission a few hours following its onset.

A follow up at 1 month (T2) and 3 months (T3) was conducted using the same assessment tools (Figure 1). At T2, the scales indicated a stable clinical condition (HAM-A = 5; HAM-D = 7), with a slight increase in both scales but no pathological score, and a worsening on both scales at T3, with a mild score in both anxiety and depression (HAM-A = 10; HAM-D = 8).

## 3. Discussion

This case describes the effect of two combined neuromodulation techniques (tDCS and rTMS in succession), which, in a short time, have shown a good efficacy in reducing symptoms in a patient with depression and generalized anxiety.

The designed protocol included a first session of tDCS (2000 µA) followed by a low frequency rTMS stimulation of the rDLPFC, and then a second session provided the same tDCS treatment as the first one, followed by a high frequency rTMS stimulation of the lDLPFC. The effectiveness of this kind of protocol has already been investigated in literature, which described the sequential bilateral high frequency rTSM of the lDLPFC and a low frequency rTMS of the rDLPFC in depression and anxiety in comorbidity [18], an accelerated bilateral theta burst stimulation (TBS) for the treatment of depression [19], and the treatment of suicide ideation with a 5 consecutive day TBS protocol [20].

The choice of DLPFC as the target area of treatment arose from the observations of common prefrontal abnormalities, both in depression and anxiety [21], with a possible laterality in the expression and regulation [22].

The use of tDCS in this case also draws from literature data, which showed that an anodal stimulation of the lDLPFC had a level A of evidence in the treatment of depression [7]; however, there was not the same level of evidence in the treatment of anxiety, where tDCS was shown to lead to a reduction in symptoms compared to placebo [23], with optimal sessions lasting 20 minutes (sessions of 20, 30 or 60 minutes have been tested) and an optimal current intensity of 2 mA [24]. However, compared to common interventions which lasted more than 10 days [8], our case involved the use of combined techniques over just 5 days of treatment, thus ensuring an intensive, brief, and effective protocol which was psychologically well-tolerated by the patient.

In any case, a near-clinical stabilization was observed one month after the end of the treatment, followed by a deterioration at three months, thus suggesting the need to reinforce the intervention with at least monthly booster sessions. Considering the patient's age, this pattern may indicate that aging could contribute to a reduced duration of treatment effects, as neuroplasticity, which underlies the efficacy of neuromodulation techniques, tends to decline with age [25].

Moreover, it is possible that the prior use of medications for the treatment of depression (such as duloxetine, quetiapine, and benzodiazepines) may have limited the neuromodulatory effects of the applied techniques [26],[27]. However, neuromodulation techniques have also emerged as augmentation strategies for the treatment of anxiety and depression in individuals who exhibit partial or poor responses to pharmacological or psychotherapeutic interventions. Our study supports the potential of non-invasive brain stimulation (NIBS) techniques as effective augmentation or combination therapies for anxiety and depression, which is in agreement with other recent studies [23],[28],[29].

## 4. Conclusions

The results of the present case are promising, and demonstrated that combining two neuromodulation techniques (tDCS and rTMS) can effectively reduce the symptoms of depression and anxiety within just a few days of treatment.

Nevertheless, this study has some limitations. First, the identification of the brain areas was not performed using MRI-guided neuronavigation, which may have slightly reduced the accuracy of the stimulation targeting. Second, as this was a single case report, the findings cannot be generalized and should be considered exploratory. Therefore, further studies are warranted to improve the statistical power, investigate the long-term durability of the clinical effects, and assess the efficacy of the protocol in more severe cases. Future randomized controlled trials (RCTs) should aim to evaluate the broader applicability and potential limitations of this combined neuromodulation approach, thus contributing to a more comprehensive understanding of its therapeutic potential.

## Use of AI tools declaration

The authors declare they have not used Artificial Intelligence (AI) tools in the creation of this article.
